# High Content Analysis of Macrophage-Targeting *Eh*PIb-Compounds against Cutaneous and Visceral *Leishmania* Species

**DOI:** 10.3390/microorganisms9020422

**Published:** 2021-02-18

**Authors:** Helena Fehling, Hanno Niss, Annika Bea, Nadine Kottmayr, Christine Brinker, Stefan Hoenow, Julie Sellau, Tim-Wolf Gilberger, Frederic Ting, Dirk Landschulze, Chris Meier, Joachim Clos, Hannelore Lotter

**Affiliations:** 1 Department of Molecular Parasitology and Immunology, Bernhard Nocht Institute for Tropical Medicine, 20359 Hamburg, Germany; niss@bnitm.de (H.N.); annika.bea@bnitm.de (A.B.); nadine-k95@web.de (N.K.); hoenow@bnitm.de (S.H.); sellau@bnitm.de (J.S.); gilberger@bnitm.de (T.-W.G.); lotter@bnitm.de (H.L.); 2 Leishmaniasis Group, Bernhard Nocht Institute for Tropical Medicine, 20359 Hamburg, Germany; brinker@bnitm.de (C.B.); clos@bnitm.de (J.C.); 3 Centre for Structural Systems Biology (CSSB), 22607 Hamburg, Germany; 4 Biology Department, Faculty of Sciences, Universität Hamburg, 22609 Hamburg, Germany; 5 Department of Chemistry, Faculty of Sciences, Universität Hamburg, 20146 Hamburg, Germany; fredericting@gmx.de (F.T.); dirk.landschulze@chemie.uni-hamburg.de (D.L.); chris.meier@chemie.uni-hamburg.de (C.M.)

**Keywords:** *Leishmania*, immunotherapy, high content screening, macrophages, drug discovery

## Abstract

An immunostimulatory glycolipid molecule from the intestinal protozoan parasite *Entamoeba histolytica* (*Eh*) and its synthetic analogs derived from its phosphatidylinositol-b-anchor (*Eh*PIb) previously showed considerable immunotherapeutic effects against *Leishmania major* infection in vitro and in vivo. Here, we describe a high content screening assay, based on primary murine macrophages. Parasites detection is based on a 90 kDA heat shock protein-specific staining, enabling the detection of several *Leishmania* species. We validated the assay using *L. major*, *L. braziliensis*, *L. donovani,* and *L. infantum* as well as investigated the anti-leishmanial activity of six immunostimulatory *Eh*PIb-compounds (Eh-1 to Eh-6). Macrophages infected with dermotropic species were more sensitive towards treatment with the compounds as their viability showed a stronger reduction compared to macrophages infected with viscerotropic species. Most compounds caused a significant reduction of the infection rates and the parasite burdens depending on the infecting species. Only compound Eh-6 was found to have activity against all *Leishmania* species. Considering the challenges in anti-leishmanial drug discovery, we developed a multi-species screening assay capable of utilizing non-recombinant parasite strains, and demonstrated its usefulness by screening macrophage-targeting *Eh*PIb-compounds showing their potential for the treatment of cutaneous and visceral leishmaniasis.

## 1. Introduction

The leishmaniases are an increasingly prevalent, poverty-related, and complex group of neglected diseases caused by infection with obligate intracellular parasites of the genus *Leishmania.* Approximately 20 *Leishmania* (*L.*) species are pathogenic to humans and are transmitted by the bite of female sandflies, e.g., *Phlebotomus* spp. and *Lutzomyia* spp. Leishmaniasis affects over 12 million people worldwide and is endemic in at least 98 tropical and subtropical countries with an estimated number of 700,000 to 1 million new cases annually [1]. Clinical symptoms can vary greatly, depending on the infecting species and host immunological factors, ranging from mild self-limiting cutaneous ulcers (cutaneous leishmaniasis—CL; caused by *L. major,* etc.) and severe, highly disfiguring mucous ulcers (mucocutaneous leishmaniasis—MCL; mainly caused by *L. braziliensis*) to visceral leishmaniasis (VL; mainly caused by *L. donovani* or *L. infantum*), which are fatal if left untreated [2].

Current chemotherapeutic options come with a number of disadvantages. The risk of severe side effects associated with toxicity and prolonged high-dose treatment regimens, as well as the emergence of drug-resistant parasites are just some of the main drawbacks of current antileishmanial drugs such as pentavalent antimonials, miltefosine, amphotericin B, paromomycin or pentamidine [3,4,5]. To overcome these limitations together with the high attrition rate observed in drug discovery pipelines, novel treatment strategies leading to new drugs are urgently needed.

The process of drug discovery is much accelerated by rapid in vitro screenings of compound libraries through automated platforms, such as high content screening (HCS) systems. So far, there are several screening assays available for *Leishmania* based on different life cycle stages and host cells [6,7,8,9,10]. Elimination of intracellular *Leishmania* can be achieved by targeting the parasite directly using established anti-leishmanial drugs. However, boosting the host immune response can also play a decisive role in eliminating *Leishmania* infections. As macrophages are the key host and sentinel cells for *Leishmania*, they constitute an optimal target for immunotherapy due to their innate immune functions [11]. Since it is well known that *Leishmania* has evolved mechanisms to subvert the immune response by reprogramming host cells such as macrophages [12], there are several studies on immunomodulatory drugs [13,14,15,16,17,18,19]. These studies mainly address the cutaneous form of leishmaniasis, with compounds such as imiquimod (Toll-like receptor TLR 7/8 agonist) [15,20] or CpG D35, an oligodeoxynucleotide containing CpG motif, which reduces the severity of cutaneous lesions by TLR9 engagement [13,14]. All the investigations of immune modulators led to the assumption, that their use in combination with conventional anti-leishmanial drugs may allow shorter treatment periods, reduce cytotoxicity, and may lower the risk of resistance.

We recently reported the immunostimulatory activity of a novel set of synthetic analogs derived from the phosphatidylinositol b anchor (PIb) of a lipopeptidephosphoglycan (LPPG) isolated from the membrane of the protozoan parasite *Entamoeba histolytica* (*Eh*). *Eh*LPPG activates macrophages by Myd88-dependend TLR or scavenger receptor ligation and a simultaneously CD1D-mediated activation of natural killer T (NKT) cells, which result in a protective Th1 immune response by the increased production of interleukin 12 (IL-12) and interferon (IFN-γ) [21,22,23]. Treatment with *Eh*PIb-compounds of murine and human macrophages infected with *L. major* in vitro resulted in a decreased parasite load. In addition, a topical application of one selected compound (Eh-1) reduced cutaneous lesions in a murine model of experimental cutaneous leishmaniasis [24].

Here, we established a multispecies HCS assay using different, non-recombinant *Leishmania* species and primary macrophages. We demonstrated its usefulness by screening the anti-leishmanial activity of a set of synthesized immunostimulatory *Eh*PIb-compounds against intracellular amastigotes of both dermotropic and viscerotropic *Leishmania* species.

## 2. Materials and Methods

### 2.1. Ethics

Donor mice for the isolation of bone-marrow derived macrophages (BMDMs) were bred and handled under pathogen-free conditions at the animal facility of the Bernhard Nocht Institute for Tropical Medicine (BNITM), Hamburg, Germany accredited by the *Behörde für Justiz und Verbraucherschutz*, Hamburg, Germany (acquisition no.: Reg.-Nr. 07/2020). Work on animals was carried out in accordance with the guidelines adhering to the NIH institutional and animal research for the care and use of laboratory animals (ARRIVE) and approved by the review board of the State of Hamburg, Germany (acquisition no.: O 014/2018).

### 2.2. Parasites

*L. major* (MHOM/SU/73/*5*ASKH) [25], *L. donovani* (BPK190) [26], *L. infantum* (MHOM/FR/91/LEM 2259, belonging to zymodeme MON-1 clone 3511) [27,28] and *L. braziliensis* (MHOM/PE/01/PER005 cl.2) [29] were routinely grown at 25 °C in a modified medium 199 (Sigma-Aldrich, Hamburg, Germany, with Hank’s salts, 20% heat-inactivated fetal calf serum, 40 mM HEPES, pH 7.4, 0.2% NaHCO_3_, 100 µM adenine, 1.2 µg/mL 6-biopterin, 10 µg/mL haem, 1x Pen/Strep/L-glutamin, and pH 7.0 (Sigma-Aldrich, Hamburg, Germany). For infection experiments, parasites were allowed to grow to the stationary phase. Promastigotes were counted using a CASY cell counter (Roche, Mannheim, Germany).

### 2.3. Mouse Primary Macrophages

Mouse primary macrophages were prepared as described previously [23,24,30,31]. Briefly bone marrow-derived macrophages (BMDMs) were isolated from the tibiae and femurs of 8–12 week old male C57BL/6J mice and cultured for 10 days in Iscove’s Modified Dulbecco’s Medium (IMDM, Sigma-Aldrich, Hamburg, Germany) supplemented with 10% heat inactivated FCS (Sigma-Aldrich, Hamburg, Germany), 5% horse serum (Sigma-Aldrich, Hamburg, Germany), 1% L-glutamine (Biochrom, Berlin, Germany), 50 µg/mL Gentamicin (Sigma-Aldrich, Hamburg, Germany), and 10% LADMAC supernatant as a source of macrophage colony-stimulating factor [31,32]. Bone marrow cells were kept in T175 ventilated cell culture flasks (Sarstedt, Nümbrecht, Germany) for 7 days and incubated at 37 °C and 5% CO_2_. Every 3 to 4 days, 50% of the medium was replaced.

### 2.4. Infection Assay

For infection, 7-day-old adherent BMDMs were harvested, washed, and seeded into tissue culture-treated 96-black-well microplates with an optically-clear cyclic olefin bottom (Cell Carrier-96 Ultra Microplates, PerkinElmer, Rodgau, Germany) at a density of 6 × 10^4^ cells in 200 µL of an IMDM^+^ medium per well. The macrophages were incubated for 72 h at 37 °C/5% CO_2_ to allow adhesion. Adherent BMDMs were infected with stationary phase promastigotes at a multiplicity of infection (MOI) ranging from 4 to 20 parasites per macrophage. For the validation of the image analysis sequence MOIs of 4:1, 8:1, and 16:1 (parasite: BMDM) for all the investigated *Leishmania* species were analyzed using the HCS system with 24 h post infection. For treatment experiments, the dermotropic species *L. major* and *L. braziliensis* were infected with a MOI of 20:1, and the viscerotropic species *L. donovani* and *L. infantum* were infected with a MOI of 8:1 and investigated 48 and 72 h post infection.

The cells were incubated post infection for 4 h and then three washing steps with warm PBS were performed in order to remove the free parasites. Internalized *Leishmania* parasites were allowed to differentiate into amastigotes for 24 h prior to being treated as described below. Macrophages infected with *L. major* were incubated at 34 °C/5% CO_2_, *L. braziliensis* at 31 °C/5% CO_2_, and *L. donovani* or *L. infantum* at 37 °C/5% CO_2_ for 24 and 48 h after addition of the compounds. The supernatants were removed, the cells were washed twice with warm PBS, and fixed with 4% paraformaldehyde. After fixation, microplates were stored at 4 °C (200 µL PBS/well) until being further processed for immunofluorescence analysis.

### 2.5. Treatment and Controls

In this study, the *Eh*PIb-compounds Eh-1, Eh-2, Eh-3, Eh-4, Eh-5, and Eh-6 used for the treatment were synthesized in a convergent synthetic route and have been shown to present anti-leishmanial activity against *L. major* [24]. Compound stocks (1 mg/mL) in DMSO were stored at −20 °C. Before use, the *Eh*PIb compound stocks were sonicated in a 37 °C preheated ultrasonic bath (Sonorex Super DK 255, Bandelin, Berlin, Germany) for 10 min and immediately used for the treatment. Infected murine macrophages were stimulated with three different concentrations (5/10/20 µM) of the *Eh*PIb-compounds as described above and analyzed after 24 and 48 h of treatment. DMSO served as a vehicle control. Amphotericin B-treated cells (1 µM, Gibco, Dublin, Ireland) as well as uninfected BMDMs were used as positive controls and infected, but untreated cells were used as a negative control.

### 2.6. Immunofluorescence Assay

Immunofluorescence staining was performed with fixed cells obtained from the infection assay. All the incubation and washing steps (5 min per every washing step) were carried out at room temperature and agitated at 300 rpm/min (IKA Shaker MTS 4, Staufen, Germany). Cells were washed twice in 0.1% Triton X-100 in PBS, then permeabilized for 15 min with 0.1% Triton X-100 and 50 mM ammonium chloride in PBS followed by incubation in a blocking solution (0.1% Triton X-100, 2% IgG-free BSA in PBS) for 30 min. Cells were stained with mouse anti-Hsp90 (90 kDA heat shock protein) [33,34] (1 h, 1:4000 in a blocking solution, 60 µL/well). Cells were washed three times and then incubated for 30 to 60 min in the dark with an anti-mouse (goat) IgG secondary antibody coupled to Alexa Fluor^®^ 647 (A-21236, Invitrogen) diluted 1:8000 and 4′,6-Diamidine-2-phenylindole dihydrochloride, dilactate (DAPI, D9564, Sigma-Aldrich, Hamburg, Germany) diluted 1:100 in a blocking solution (60 µL/well). After washing the cells three times, 200 µL of PBS were added to each well and the microplates were stored at 4 °C in the dark before image acquisition.

### 2.7. Image Acquisition

Images were acquired using the automatic Opera Phenix™ Nipkow confocal high-content screening (HCS) system (PerkinElmer, Rodgau, Germany) and a 20× water objective lens (NA 1.0; binning 1). Fluorescent images (15 fields per well) were recorded with DAPI channel (excitation: 405 nm/emission 435–480 nm) for the detection of macrophage nuclei and Alexa 647 channel (excitation: 640 nm/emission 650–760 nm) for visualization of anti-Hsp90-stained *Leishmania* parasites and macrophage cytoplasm. For every field in a well, a stack of six planes was set up with an overall height of 5.0 µm ranging from the first plane at −11.0 µm and the last plane at −6.0 µm with a distance of 1.0 µm. An approximate confluency of 70–80% cells was obtained per well with these settings, resulting in adequate numbers of macrophages (6000–8000) for quantification.

### 2.8. Image and Data Analysis

The acquired images were analyzed using a custom designed image analysis sequence (Table S1) of the Harmony software (version 4.6; PerkinElmer) based on the software’s integrated analysis algorithm building block approach.

The identification of host cells was primarily based on the detection of nuclei (DAPI; method: B; area: >40 µm^2^) but also of the cytoplasm (Alexa 647; method: A) By calculating intensity and morphology properties. Cells whose nuclei or cytoplasm was partly outside the field of view were excluded from the analysis, as they were considered not to be representative.

For the identification of intracellular signals (Alexa 647) referred to as spots within the analysis algorithm, the area of the cytoplasm was defined as the region of interest (ROI) to achieve a high sensitivity first. To discriminate intracellular spots corresponding to *Leishmania* parasites from false-positive intracellular spots, a very selective method was chosen based on the calculation of spot properties (intensity and morphology) to achieve a specific classification of objects. The detailed script of the image analysis sequence is shown in the Supplementary Materials, Table S1.

The main outputs (per well) generated with the image analysis sequence were (i) total number of macrophages to determine the viability of host cells after drug exposure based on their adhesive capability; (ii) total number of *Leishmania*-infected macrophages as well as, (iii) percentage of infected macrophages to determine the overall infection rate. For the detection of the parasite burden, the total number of *Leishmania* parasites was taken into account. The number of *Leishmania* parasites per infected macrophage was also recorded.

To judge the assay and image analysis quality, Z’ values were determined for each microplate to achieve a good signal-to-background ratio. Z’ values above 0.3 were considered significant for cellular screening [35], while microplates with lower Z’ values were excluded from the study.

$$Z’=1-\;\frac{3*\left(stddev\left(\mathrm{Negative}\;\mathrm{Control}\right)+stddev\left(\mathrm{Positive}\;\mathrm{Control}\right)\right)}{\left|mean\left(\mathrm{Negative}\;\mathrm{Control}\right)-\;mean\left(\mathrm{Positive}\;\mathrm{Control}\right)\right|}=1-\frac{3}{SNR}$$

Quantitative values obtained from the Harmony software were exported to Microsoft Excel for further analysis. Data were normalized to the negative control (infected, untreated BMDMs) and expressed as a percentage of the negative control (POC).

### 2.9. Statistical Analysis

Percentages of the readout parameters viability, infection rate, and parasite burden were compared between the infected vehicle control and treated cells by the unpaired Student´s t-test. Differences were considered to be significant if *p*-values were as follows: * *p* < 0.05; ** *p* < 0.01; *** *p* < 0.001; **** *p* < 0.0001. The normal distribution of the data was confirmed using the Shapiro-Wilk test as part of the GraphPad Prism statistic software version 8.0.2. Dose-response data ((inhibitor) vs. normalized response) were also processed with the GraphPad Prism using nonlinear curve fitting (Supplementary Table S2).

## 3. Results

### 3.1. Development of a High Content Screening Assay for Cutaneous and Visceral Leishmania Species

**Figure 1:**
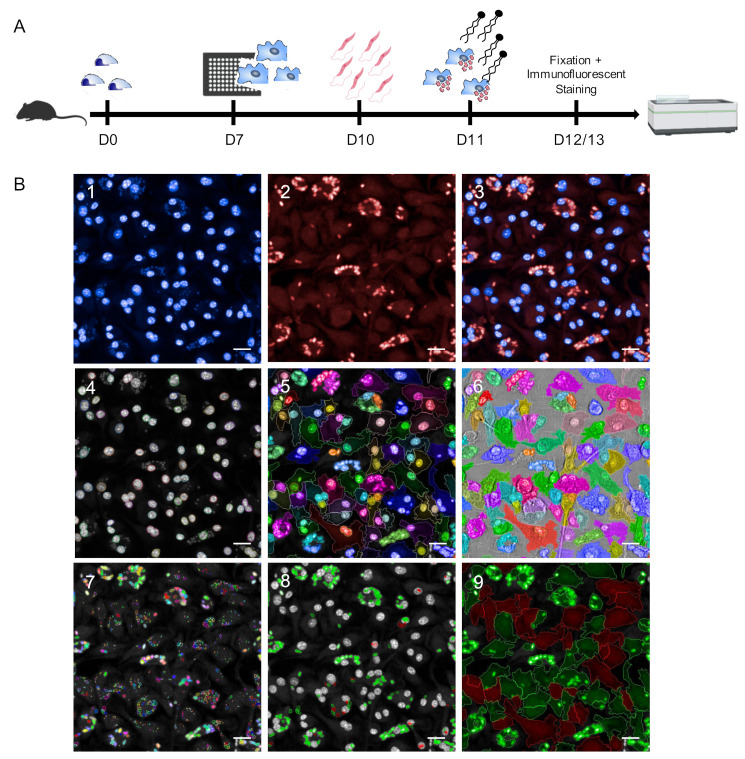
Schematic representation of the *Leishmania ssp.* screening assay. (**A**) Experimental pipeline. D0: Isolation of mouse bone marrow-derived cells; D7: Harvest and seeding of mouse primary macrophages into CellCarrier-96 Ultra Microplates; D10: Infection with *Leishmania* (*L.*) parasites (4 h); D11: Adding immunostimulatory *Eh*PIB-compounds to the cell cultures for 24 (D12) or 48 h (D13) with subsequent fixation and immunofluorescent staining of the cells followed by image acquisition and analysis using the Opera Phenix high-content screening (HCS) system. (**B**) Parameters for HCS analysis and output. (B1–3) Representative input images of bone-marrow derived macrophages (BMDMs) (DAPI; 405 nm) infected with *L. infantum* (multiplicity of infection (MOI) 8:1) (Hsp90 staining; Alexa 647; 640 nm). (B4–6) Object segmentation of macrophages based on intensity and morphology properties of nuclei detection (B4; DAPI; >40 µm^2^) and cytoplasm detection (B5; Alexa 647). (B7–8) Detection of immuno-stained intracellular *L.* parasites. B7) Detection of all spots within the region of interest defined as cell cytoplasm (B5). (B8) Selection of correct spots (green = L.-parasites, >4 µm^2^) and discarded spots (red) based on intensity and morphology properties. (B9) Output population *L.*-infected macrophages (green) and non-infected macrophages (red) based on merged images obtained from cell segmentation (B4–6) and parasite detection (B7–8). Scale bar, 20 µM. Figure 1A was created with a licensed version of BioRender.com.

In this work, we established an HCS assay for the reliable quantification of different *Leishmania* spp. using primary murine macrophages as parasite host cells. After 7 days of differentiation from murine bone marrow progenitors, BMDMs were harvested, seeded into 96-well plates, and infected with stationary phase promastigotes on day 10. At 24 h post infection, immunostimulatory *Eh*PIb-compounds were added to the cell cultures for 24 and 48 h followed by fixation, immunofluorescent staining, and image acquisition using the Opera Phenix HCS system (Figure 1A).

As *Leishmania* ssp. present different temperature tolerances for intracellular persistence and multiplication [10,36], infection with dermotropic species was performed at 31 °C (*L. braziliensis*) or 34 °C (*L. major*) (Figure S1) and infection with viscerotropic species was performed at 37 °C.

For microscopy-based high content methodologies the capacity to detect and discriminate between objects is crucial. To achieve an appropriate object segmentation, we employed DNA and anti-Hsp90 staining in our approach (Figure 1(B1–3)) to distinguish macrophages and *Leishmania* parasites (Figure 1(B4–9)). For the detection of macrophages, both nuclei (DAPI) and cytoplasm (anti-Hsp90) were taken into account (Figure 1(B4–6)). This approach allows accurately defining the boundary of primary macrophages. Hence, an exact region of interest (ROI) with heterogenous morphologies can be identified which is not based on the assumption that nuclei are located in the center of the cells, to avoid inaccurate results working with primary cells and not with homogenous macrophage cell lines. In most HCS assays, the detection of *Leishmania* parasites is based on DNA staining [8,9,10] or transgenic parasites expressing fluorescent proteins [6,7,37,38]. The use of DNA staining can lead to inaccurate results as not only the parasitic nuclear DNA is stained, but also the mitochondrial kinetoplast DNA. Non-specifically stained spots that may appear in uninfected cells due to cytosolic accumulations of host cells RNA [39] also represent a possible source of inaccuracy. Although using transgenic parasites simplifies HCS campaigns and accelerates drug discovery, their use of transgenic laboratory strains limits the testing of field isolates. To overcome these issues, we have used *Leishmania*-specific anti-Hsp90 staining for the detection of intracellular amastigotes [33] to discriminate between infected and non-infected cells (Figure 1(B7–9)). The complete image analysis sequence established in the Harmony software is shown in Supplementary Table S1.

**Figure 2:**
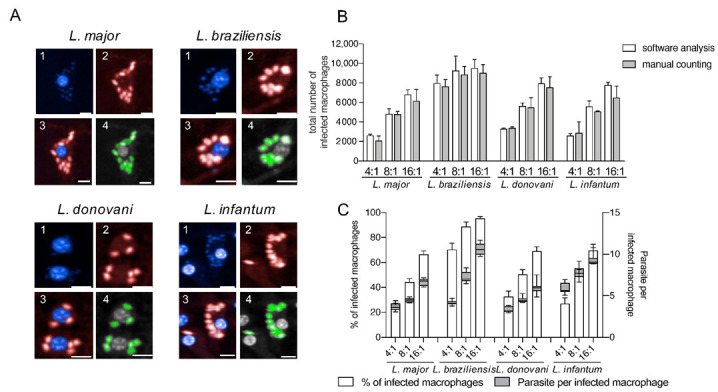
Detection of four different *Leishmania* species using an in vitro high-content screening assay. (**A**) Representative images illustrating the infection of BMDMs (1; nuclei detection; blue; DAPI) with *Leishmania* ssp. (2; parasite detection; red; Hsp90 staining) causing diseases with different clinical manifestations: *L. major* (cutaneous), *L. braziliensis* (mucocutaneous), *L. donovani* (visceral) and *L. infantum* (visceral). Images were acquired with an Opera Phenix confocal microscope (3; overlay 1 + 2), and analyzed using a custom designed image analysis sequence (Harmony software) to detect different *Leishmania* ssp. (4; green = positive selected parasites). Scale bar 10 µM. (**B**) Comparison of image analysis sequence with manual counting. Total number of infected macrophages obtained with the automated protocol was compared with manual counting (15 fields of two wells in each MOI condition). (**C**) Percentage of infected macrophages and the number of parasites per infected macrophage (24 h post infection) detected with the custom designed image analysis sequence depending on the MOI. Values represent the mean with SD (*n* = 8, per each MOI tested).

This approach reliably detects different *Leishmania* ssp. (Figure 2) without optimizing the image analysis settings for each species to obtain appropriate cell segmentation and parasite detection.

As representative *Leishmania* parasites for dermotropic and viscerotropic species, *L. major, L. braziliensis, L. donovani,* and *L. infantum* were used in this work for infection experiments (Figure 2A). To validate the image analysis sequence for all tested *Leishmania* species, the total number of infected macrophages obtained by the automated HCS protocol was compared with a manual count on the acquired images (15 fields of two representative wells for each MOI and each species) and no significant differences were registered (Figure 2B). The infection rates and the number of parasites per infected cell obtained by the image analysis sequence also increases proportionally with the MOI (Figure 2C). Therefore, we conclude that our HCS assay is adequate to perform a phenotypic, multispecies screening for the analysis of anti-leishmanial efficacy of the lately described immunostimulatory *Eh*PIB-compounds [24].

### 3.2. Viability of Leishmania-Infected Macrophages after Treatment with Immunostimulatory EhPIb-Compounds

**Figure 3:**
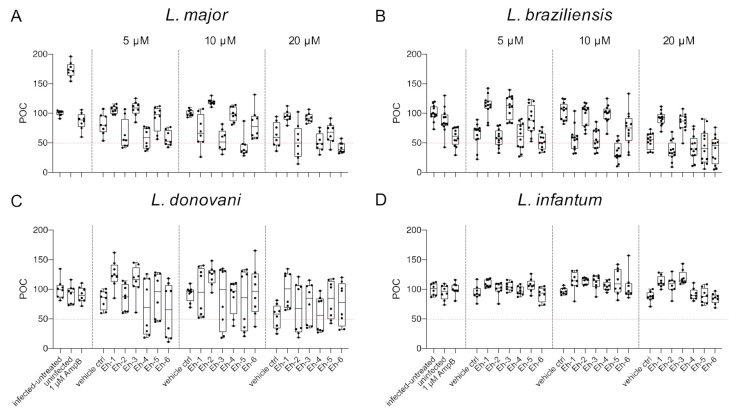
Macrophage viability post treatment with *Eh*PIb-compounds. BMDMs were infected with (**A**) *L. major* (MOI 20:1), (**B**) *L. braziliensis* (MOI 20:1), (**C**) *L. donovani* (MOI 8:1) or (**D**) *L. infantum* (MOI 8:1) and 24 h post infection treated with *Eh*PIb-compounds in different concentrations (5, 10, 20 µM) for another 48 h. Infected and untreated BMDMs (negative control), as well as uninfected, 1 µM Amphotericin B (AmpB), and DMSO (vehicle control) treated cells were used as controls. Data are expressed as boxplots with whiskers from minimum to maximum of the percent of negative control (POC) of total BMDMs. Compounds were considered toxic with a survival rate of BMDMs below 50% (red line). Analyzed data are based on either two or three independent experiments with *n* = 8–12.

To analyze the impact of synthetic *Eh*PIb analogs on macrophage viability, the total number of macrophages was determined using the custom made HCS image analysis sequence. BMDMs were infected with *L. major* (MOI 20:1), *L. braziliensis* (MOI 20:1), *L. donovani* (MOI 8:1), and *L. infantum* (MOI 8:1) and incubated with the *Eh*PIb-compounds Eh-1, Eh-2, Eh-3, Eh-4, Eh-5, and Eh-6 for 48 h at different concentrations (5/10/20 µM). Infected and untreated BMDMs as well as uninfected, 1 µM AmpB-treated and vehicle controls (DMSO) served as controls. A survival rate of BMDMs below 50% was considered to have a toxic effect on the host cells (Figure 3).

Varying levels of macrophage viability were observed across the different *Leishmania* species and a dose-dependency was not clearly evident. Concentrations < 1% of DMSO seemed to be well tolerated by the cells, whereas higher concentrations (20 µM = 1.6% DMSO) resulted in reduced macrophage survival (*L. major*: −36.76% ± 7.63; *L. braziliensis*: −46.63% ± 5.49; *L. donovani*: −46.04% ± 9.35). Interestingly, the treatment with the *Eh*PIb-compounds had a similar effect on cell viability in murine macrophages infected with the dermotropic *Leishmania* species *L. major* (Figure 3A) and *L. braziliensis* (Figure 3B) depending on the compound used. Eh-5 exhibited the highest toxicity at 10 µM (*L. major*: −56.22% ± 6.8; *L. braziliensis*: −65.97% ± 5.6). In contrast, the treatment of BMDMs infected with the viscerotropic species *L. donovani* (Figure 3C) resulted in a better survival rate of macrophages, whereas all the compounds induced no toxicity in BMDMs infected with *L. infantum* (Figure 3D). In conclusion, the viability of host cells indicated by their adherence after the treatment with the immunostimulatory *Eh*PIb-compounds seem to be dependent on the infecting species.

### 3.3. In Vitro Activities of EhPIb-Compounds against Different LEISHMANIA Infections

**Figure 4:**
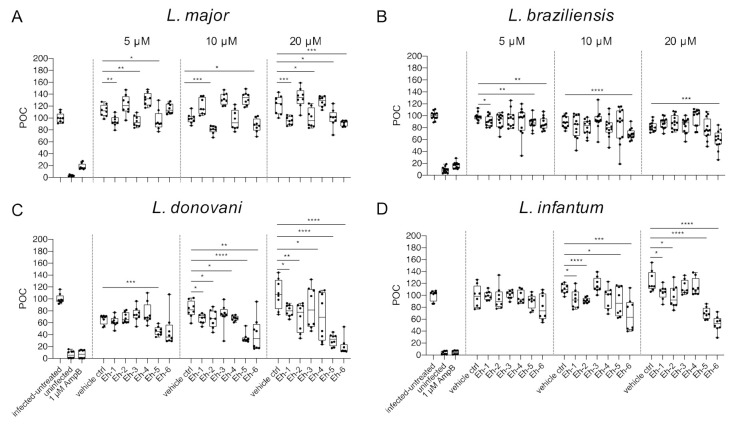
Infection rate post treatment with *Eh*PIb-compounds. BMDMs were infected with (**A**) *L. major* (MOI 20:1), (**B**) *L. braziliensis* (MOI 20:1), (**C**) *L. donovani* (MOI 8:1) or (**D**) *L. infantum* (MOI 8:1) and 24 h post infection treated with *Eh*PIb-compounds in different concentrations (5, 10, 20 µM) for another 48 h. Infected and untreated BMDMs (negative control), as well as uninfected, 1 µM Amphotericin B (AmpB), and DMSO (vehicle control) treated cells were used as controls. Data are expressed as boxplots with whiskers from minimum to maximum of the percent of negative control (POC) of total BMDMs and the treated samples were compared to the vehicle control. Analyzed data are based on either two or three independent experiments with *n* = 8–12: * *p* < 0.05; ** *p* < 0.01; *** *p* < 0.001; **** *p* < 0.0001 (unpaired Student’s *t*-test).

We previously reported that the immunostimulatory *Eh*PIb-compounds reduce parasite loads in *L. major*-infected BMDMs and human THP1 cells based on molecular detection using real-time PCR by the duplex TaqMan PCR [24] and found a considerable anti-leishmanial activity for the majority of the synthetic molecules. Here, we investigated the anti-leishmanial activity of the *Eh*PIb-compounds (5/10/20 µM) against *L. major*-, *L. braziliensis*-, *L. donovani*-, and *L. infantum*-infected BMDMs using the HCS technology. First, we analyzed the impact on the infection rate 48 h post treatment compared to the vehicle control (Figure 4).

The synthetic *Eh*PIb-compounds Eh-1 (5, 20 µM), Eh-2 (10 µM), Eh-3 (5, 20 µM), Eh-5 (5, 20 µM), and Eh-6 (10, 20 µM) significantly reduced the percentage of *L. major*-infected macrophages, whereas Eh-4 had no effect on the infection rate at any concentration (Figure 4A). The treatment with Eh-6 caused a significant reduction of *L. braziliensis*-infected macrophages at all concentrations tested, whereas Eh-1 and Eh-5 only reduced the infection rate at a concentration of 5 µM (Figure 4B). The majority of the immunostimulatory *Eh*PIb-compounds showed significant effects on the infection rate of *L. donovani*-infected macrophages except Eh-3 (Figure 4C). Eh-1, Eh-2, Eh-5, and Eh-6 significantly reduced the percentage of *L. infantum*-infected macrophages at concentrations of 10 and 20 µM, while the treatment with Eh-3 and Eh-4 showed no anti-leishmanial effects (Figure 4D). Eh-1 and Eh-6 were the only compounds that significantly reduced the infection rate in all *Leishmania* species. A clear dose-dependency, due to the lipid structure of the synthetic analogs, which presumably leads to micelle formation, made an accurate IC_50_ determination difficult (Table S2). For the treatment with compound Eh-6 an IC_50_ [95% CI] of 4.685 [2.91 to 7.16] was determined for the experimental infection with *L. braziliensis* and an IC_50_ [95% CI] of [2.294 1.20 to 3.73] was determined for the experimental infection with *L. donovani*.

**Figure 5:**
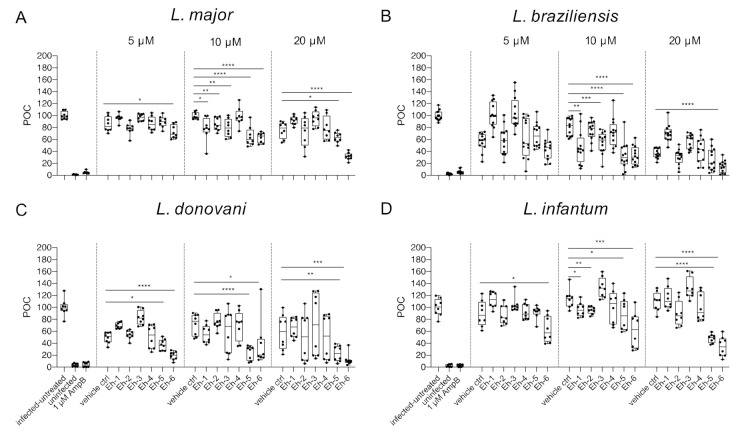
Parasite burden post treatment with *Eh*PIb-compounds. BMDMs were infected with (**A**) *L. major* (MOI 20:1), (**B**) *L. braziliensis* (MOI 20:1), (**C**) *L. donovani* (MOI 8:1) or (**D**) *L. infantum* (MOI 8:1) and 24 h post infection treated with *Eh*PIb-compounds in different concentrations (5, 10, 20 µM) for another 48 h. Infected and untreated BMDMs (negative control), as well as uninfected, 1 µM Amphotericin B (AmpB), and DMSO (vehicle control) treated cells were used as controls. Data are expressed as boxplots with whiskers from minimum to maximum of the percent of negative control (POC) of total BMDMs and the treated samples were compared to the vehicle control. Analyzed data are based on either two or three independent experiments with *n* = 8–12: * *p* < 0.05; ** *p* < 0.01; *** *p* < 0.001; **** *p* < 0.0001 (unpaired Student’s *t*-test).

We next examined the parasite burden at 48 h post treatment with the immunostimulatory *Eh*PIb-compounds by comparing the total number of detected *Leishmania* parasites in *Eh*PIb-treated versus vehicle control (Figure 5).

With the exception of Eh-4, all the compounds caused a significant reduction of the overall parasite burden at a concentration of 10 µM in *L. major*-infected BMDMs (Figure 5A). For BMDMs infected with *L. braziliensis,* the strongest decrease of the parasite burden was found at concentrations of 10 µM with the compounds Eh-1, Eh-3, Eh-5, and Eh-6, whereas, Eh-2 and Eh-4 showed no anti-leishmanial activity (Figure 5B). Although the treatment with most *Eh*PIb-compounds reduced the infection rate of *L. donovani*-infected BMDMs, the parasite burden was only significantly reduced after the addition of Eh-5 and Eh-6 (Figure 5C). Eh-5 and Eh-6 also induced the most significant reduction of the parasite burden in *L. infantum*-infected macrophages, but Eh-1 and Eh-2 also led to a significant decrease of the parasite burden at concentrations of 10 µM (Figure 5D). Eh-6 was the only compound that significantly reduced the parasite burden caused by all tested *Leishmania* species at the majority of tested concentrations.

**Figure 6:**
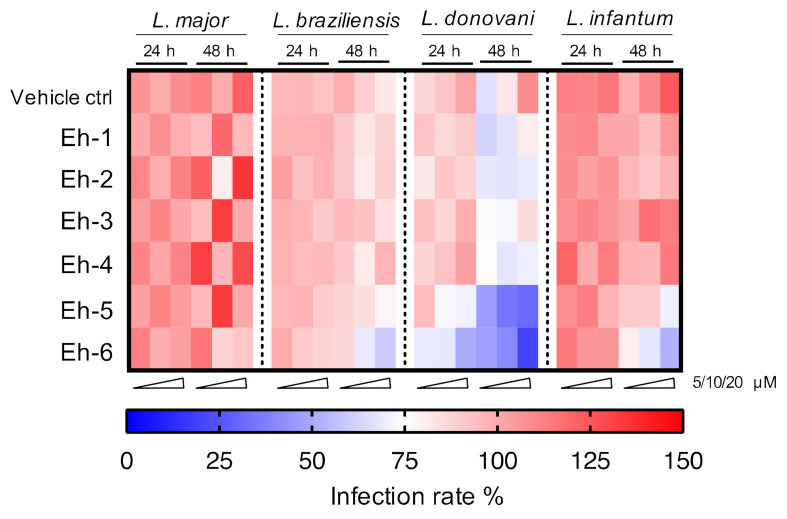
Heatmap of screening results for the *Eh*PIb-compound activity against different *Leishmania* species. BMDMs were infected with *L. major* (MOI 20:1), *L. braziliensis* (MOI 20:1), *L. donovani* (MOI 8:1) or *L. infantum* (MOI 8:1) and 24 h post infection treated with *Eh*PIb-compounds in different concentrations (5, 10, 20 µM) for another 24 and 48 h. The colour scale of the heatmap illustrates the mean normalized infection rate (%) from two to three independent experiments in relation to controls: 100% infected BMDMs (red = no activity), 75% infected BMDMs (blank = moderate activity), and zero % infected BMDMs (blue = strong activity).

Six immunostimulatory *Eh*PIb-compounds were evaluated over treatment periods of 24 and 48 h against the two dermotropic species *L. major* and *L. braziliensis*, as well as against the viscerotropic species *L. donovani* and *L. infantum*. The overall activity against the different species is shown in a heat map in Figure 6.

Most compounds presented activity after a 48 h incubation period. Macrophages infected with viscerotropic species show a higher sensitivity to the compounds leading to a better clearance of the parasites.

In summary, this first screening of *Eh*PIb-compounds against different *Leishmania* species shows a considerable anti-parasitic effect, but also demonstrates a high species-specificity that may reflect different mechanisms by which the parasites circumvent their elimination inside macrophages.

## 4. Discussion

Immunotherapy is an increasingly important treatment strategy for a broad spectrum of diseases. In infectious diseases in particular, host-response enhancing drugs can avoid many of the problems caused by pathogen-targeting drugs, by acting directly on host molecules such as reactive oxygen species (ROS), and nitric oxide (NO) or TLR dependent pathways which are critical for pathogen invasion, survival and/or replication, but redundant for the host. Additionally, host-targeting molecules are likely to have a less chance of selecting for resistances and may reduce toxic side effects. Therefore, we screened a set of six immunostimulatory *Eh*PIb-compounds against the four clinically relevant species *L. major*, *L. braziliensis*, *L. donovani,* and *L. infantum* using a novel high content screening assay. We identified one hit compound with a broad activity range at 10 µM of concentration against both dermotropic and viscerotropic *Leishmania* species.

In early drug discovery, HCS assays are the state-of-the-art strategy to identify clinically relevant hit compounds. Previously developed HCS assays for *Leishmania* often use immortalized cell lines, or genetically modified parasites or axenic amastigotes expressing reporter genes such as green fluorescence protein (GFP) or DsRed2 molecule to facilitate parasite detection [6,7,8,9,10,37]. In order to mimic natural infection as closely as possible in an in vivo infection, the present assay was established with primary murine macrophages and is easily transferrable to primary human macrophages (A.B., unpublished). Although primary cells are rather sensitive, they have a normal cell morphology and maintain many of the important markers and functions seen in vivo. Whereas, cell lines often differ genetically and phenotypically from their tissue origin and show altered morphology, they are easier to culture and more stable. When performing phenotypic screenings of immunostimulatory compounds, targeting host cells and not the parasites, data from primary cells better reflect the in vivo scenario. Similarly, the use of genetically modified parasites expressing fluorescent proteins may not reflect natural infection as the reporter genes may interfere with drug screening results by decreasing fitness [8,9,40]. Moreover, the use of reporter transgenes precludes the screening with larger panels of parasite species/isolate, and is problematic with *L. braziliensis* [41]. The use of axenic amastigotes, i.e., free-living, amastigote-like cells, in screening will completely miss compounds that stimulate host cell leishmanicidal activity [9]. Hence, the use of axenic amastigotes for drug screening campaigns raises major concerns, also since there are profound differences to *bona fide* amastigotes with respect to gene expression, morphology, and proliferation [7,42,43].

To improve specificity and sensitivity of intracellular parasite detection in our HCS assay, we used an anti-Hsp90 staining. Although this approach is more time-consuming, it allows the use of any non-recombinant *Leishmania* strain without modifying the image analysis parameters. The protocol developed in this study can also be used for determining the infectivity and intracellular survival of gene replacement mutants or other genetic variations of *Leishmania*.

In this study, we applied the novel HCS assay to perform a multispecies activity screening of the previously described, anti-leishmanial *Eh*PIb-compounds. We had already shown that the immunostimulatory glycolipid *Eh*LPPG and its synthetic analogs induce a protective cytokine response leading to anti-parasitic effects during in vitro infections and in two different in vivo models of *L. major* infection [21,23,24]. This unique and dual mode of immune activation by *Eh*LPPG is elicited by TLR2/TLR6 engagement and Myd88 signalling, which induces production of pro-inflammatory cytokines such as IL 12, and by the simultaneous uptake of the molecule leading to processing and loading onto CD1d molecules in the late endosomes of antigen-presenting cells. The latter results in the presentation to invariant NKT cells and elicits a strong immune response [21]. Both of these pathways are relevant for optimal NKT cell activation, which plays a crucial role in early *Leishmania* infection since cytokine production might influence the disease outcome [44]. The analogs were found to induce the production of substantial IFNγ and IL-4 titers by human NKT cells and of TNFα by human PBMCs in vitro [23]. Upon treatment with Eh-1, an increased serum level of protective cytokines (TNFα, IFNγ, IL-23) in *L. major* infected C57BL/6 mice was observed [24]. Resistance to *Leishmania* infection correlates with a Th1-type immune response, with macrophages as the key host cells for parasite clearance. We previously showed that macrophages are activated and polarized via the classical pathway towards M1 macrophages upon the Eh-1 treatment, as arginase and IL-4 reduction was observed in vitro and the number of arginase expressing M2 macrophages was also higher in vivo in non-treated animals [24]. This immunomodulation induced by the native molecule and the synthetic analogs is likely to affect the clinical outcome of leishmaniasis, since M1 macrophages produce inducible nitric oxide synthase, iNOS, which converts L-arginine to NO and mediates to parasite killing. By contrast, M2 macrophages induce arginase, which hydrolyses L-arginine to ornithine, resulting in parasite survival [45].

Due to the structural similarities with *Eh*LPPG, we expect the synthetic *Eh*PIb-compounds to activate macrophages and NKT cells in a similar manner. This actually represents a clear limitation of all in vitro screening assays, as the complex interplay of different types of immune cells cannot be mimicked with macrophages alone.

The synthetic *Eh*PIb-compounds all consist of two short C16:0 fatty acids and vary in their phosphatidylinositol and their glycerol moieties. The hydrophilic and hydrophobic structures of the fatty acids may lead to micelle formation, allowing insertion into the lipid bilayer of eukaryotic cell membranes inducing rupture and thus, cytotoxicity [46]. We previously demonstrated that the *Eh*PIb-compounds only induced negligible cytotoxicity against uninfected murine splenocytes and human peripheral blood lymphocytes [24]. Here, we used the output of the HCS assay to analyze their impact on the viability of murine macrophages infected with two dermotropic and two viscerotropic *Leishmania* species. Interestingly, macrophages infected with the cutanotropic species *L. major* and *L. braziliensis* were generally more sensitive to the *Eh*PIb-compounds, and the treatment induced stronger cytotoxic effects in the host cells compared to macrophages infected with *L. donovani* or *L. infantum*. Eh−5 exhibited the highest toxicity at 10 µM after infection with dermotropic species (*L. major*: −56.22% ± 6.8; *L. braziliensis*: −65.97% ± 5.6) but only moderate to negligible toxicity infected with *L. donovani* (−19.40% ± 19.09) or *L. infantum* (12.79% ± 8.518) (Figure 3). We speculate that the molecular interaction between the dermotropic species and their host cells, which normally increases the life span of infected macrophages upon infection [47,48,49], may be affected by the treatment with the immunostimulatory *Eh*PIb-compounds in a different manner compared to the viscerotropic species. It is known that the infection of macrophages with *L. donovani* increases the viability of host cells in the absence of exogenous growth factors [47], whereas the prevention of programmed cell death in BMDMs infected with *L. major* is associated with a repression of mitochondrial release of cytochrome c [48].

These species-specific differences also reflected in the impact of the *Eh*PIb-compounds on the infection rates and parasite burdens. Less macrophages remained infected with viscerotropic species under the treatment, compared to the experimental infection models with the dermotropic species (Figure 4). Conversely, parasite burdens were reduced more efficiently by most compounds in macrophages infected with *L. major* and *L. braziliensis*, indicating a correlation with the typical clinical manifestations (Figure 5). Out of the six *Eh*PIb-compounds, only Eh-6 had a significant effect on the infection rates and the parasite burdens for all tested species. Consistent with earlier results, a clear dose-dependency was not observed as well as a large scatter of the individual biological samples, precluding an IC_50_ measurement (Table S2). Moreover, the determination of EC_50_ in BMDMs and the calculation of the selectivity index are not yet correctly feasible according to the current structure of the molecules. We suspect that one reason for this could be that certain analogues tend to form micelles quickly and spontaneously in an aqueous solution due to their hydrophobic properties, despite the extensive ultrasound treatment before the compounds are used. This micelle formation may lead to an unspecific immune activation, which may even promote the survival of intracellular parasites. Therefore, only an IC_50_ determination was possible for compound Eh-5 (IC_50_ 0.93 [95% CI 2.91 to 7.16]) and Eh-6 (IC_50_ 2.294 [95% CI 1.20 to 3.73]) in the experimental infection with *L. donovani* as well as for compound Eh-6 in the experimental infection with *L. braziliensis* (IC_50_ 4.685 [95% CI 2.91 to 7.16]). One way to avoid micelle formation would be to incorporate the analogues into suitable nanocarriers, e.g., liposomes. However, as such procedures are very elaborate and costly, and the stability of such formulations is limited, this should only be tested with the most effective compounds.

## 5. Conclusions

The assay presented here is a robust and reliable method for the identification of therapeutics against various *Leishmania* ssp. on a small scale. It allows the quantification of intracellular amastigotes of different and non-recombinant *Leishmania* strains and is suitable to determine the infectivity and intracellular survival of gene replacement mutants or other genetic variations of *Leishmania*.

Using the newly established protocol, synthetic *Eh*PIb-compounds were shown to elicit a substantial anti-parasitic immune response in macrophages directed against dermotropic and viscerotropic *Leishmania* species. Nevertheless, further studies are also needed to develop a suitable formulation of the synthetic analogs and to gain deeper insights into the mechanisms of action of the *Eh*PIb-compounds.

## 6. Patents

For the therapeutical synthetic analogs Eh-1 to Eh-4, patent applications titled “NEW IMMUNOSTIMULATORY COMPOUNDS” are pending in Europe (EP15770891.8), Brazil (BR112017006177-5), and India (IN201747014564).

## Data Availability

Data is contained within the article and Supplementary Material.

## Supplementary Materials

The following are available online at https://www.mdpi.com/2076-2607/9/2/422/s1. Figure S1: Influence on the assay temperature for infection with cutaneous *Leishmania* species. Percentage of infected bone-marrow derived macrophages (BMDMs) infected with (A) *L. major* (37, 34 °C) or (B) *L. braziliensis* (34, 31 °C) at different incubation temperatures and different multiplicities of infection (MOIs; 4:1/8:1/10:1/15:1/20:1) after 48 and 72 h post infection. Data are expressed as boxplots with whiskers from the minimum to maximum of percent of infected macrophages (*n* = 8); Table S1: Image analysis sequence established in the Harmony software; Table S2: Structures of *Eh*PIb-compounds and IC_50_ determination.
