# Supplementary material for: High Content Analysis of Macrophage-Targeting EhPIb-Compounds against Cutaneous and Visceral Leishmania Species
Source: Microorganisms. 2021 Feb 18;9(2):422. doi: 10.3390/microorganisms9020422 (PMC7923059; doi:10.3390/microorganisms9020422)
Supplement: Supplementary file 1 [file microorganisms-09-00422-s001.pdf]

Supplementary tables

Table S1: Image analysis sequence established in Harmony software.

Analysis building block	Objective/ Specification	Parameters		
FIND NUCLEI	**Image segmentation:** Determines host cell nuclei	Channel:<br>ROI:<br>Method:<br>Common Threshold:<br>Area:<br>Split Factor:<br>Individual Threshold:<br>Contrast:<br>Output Population:	DAPI<br>None<br>B<br>0.02<br>> 40 µm²<br>16.9<br>0.14<br>> -0.71<br>Macrophages	
FIND CYTOPLASM	**Image segmentation:** Identifies host cell cytoplasm and defines single cells	Channel:<br>Nuclei:<br>Method:<br>Individual Threshold:	Alexa 647<br>Macrophages<br>A<br>0.06	
CALCULATE INTENSITY PROPERTIES	**Define regions of interest:** Quantifies properties in regions and removes border objects	Channel:<br>Population:<br>Region:<br>Method:<br>Mean Property Prefix:	DAPI<br>Macrophages<br>Nucleus<br>Standard<br>Nucleus DAPI	Alexa 647<br>Macrophages<br>Cell<br>Standard<br>Cell Alexa 647
CALCULATE MORPHOLOGY PROPERTIES		Population:<br>Region:<br>Method:<br>Property Prefix:	Macrophages<br>Nucleus<br>Standard (area/ roundness)<br>Nucleus	
FIND SPOTS	**Image Segmentation:** Identifies intracellular spots within the region of interest	Channel:<br>ROI:	Alexa 647<br>Macrophages	
		ROI Region:<br>Method:<br>Detection Sensitivity:<br>Splitting Coefficient:<br>Calculate Spot Properties<br>Output Population:	Cell<br>B<br>0.11<br>0.844<br><br>Spots	
CALCULATE MORPHOLOGY PROPERTIES	**Define properties of spots:** Quantification and calculation	Input Population:<br>Region:	Spots<br>Spot	
		Method:<br>Output Property Prefix:	Standard (area / roundness/ width/ length/ ratio width to length)<br>Spot	
		Input Channel:<br>Population:	Alexa 647<br>Spots	DAPI<br>Spots

<table>
<tr><td rowspan="2">CALCULATE INTENSITY PROPERTIES</td><td rowspan="2">of intracellular parasites</td><td>Region:
Method:
Output: Property Prefix:</td><td>Spot
Standard (mean/ standard deviation/ coefficient of variance/ median)
Intensity Spot Alexa 647</td><td>Spot
Standard (mean)
Intensity Spot DAPI</td></tr>
<tr></tr>
<tr><td>CALCULATE PROPERTIES</td><td></td><td>Population:
Method:
Formula:
Variable A:
Variable B:
Output Property:</td><td colspan="2">Spots
By Formula
A/B
Spot Alexa 647 mean
Spot DAPI mean
ALEXA/ DAPI intensity ratio</td></tr>
<tr><td rowspan="6">SELECT POPULATION I</td><td rowspan="6">Identify intracellular Leishmania parasites: Selects parasites from false-positive spots</td><td>Input Population:
Method:
Number of Classes:</td><td colspan="2">Spots
Linear Classifier
2</td></tr>
<tr><td>Relative Spot Intensity
Corrected Spot Intensity
Uncorrected Spot Peak Intensity
Spot Contrast
Spot Background Intensity
Spot Area [px²]
Region Intensity
Spot to Region Intensity
Spot Area [µm²]</td><td colspan="2">Spot Roundness
Spot Width [µm]
Spot Length [µm]
Spot Ratio Width to Length
Spot Alexa 568 Mean
Spot DAPI Mean
ALEXA/DAPI intensity ratio
Intensity Surrounding Alexa 568 Mean
Intensity Surrounding Alexa 568 Median</td></tr>
<tr><td>Output Population A:
Output Population B:</td><td colspan="2">Likely Leishmania
False-positive</td></tr>
<tr><td>Population:</td><td colspan="2">Spots</td></tr>
<tr><td>Method:
Likely Leishmania:
Output Population:</td><td colspan="2">Filter by property
>0
Spots selected</td></tr>
<tr><td>Population:
Method:
Spot Area [µm²]:
ALEXA/DAPI intensity ratio:
Spot Area [µm²]:
Corrected Spot Intensity:
Boolean Operations:</td><td colspan="2">Spots selected
Filter by Property
> 4
> 0.3
< 35
> 100 (Staining-dependent)
F1 and F2 and F3 and F4</td></tr>
<tr><td>CALCULATE PROPERTIES</td><td>Macrophages and parasites are brought into relation</td><td>Population:
Method:
Related Population:
Number of Leishmania
Output: Property Suffix:</td><td colspan="2">Macrophages
By related population
Leishmania

Per Cell</td></tr>
<tr><td></td><td></td><td>Population:
Method:</td><td colspan="2">Macrophages
Filter by Property</td></tr>
</table>

<table>
<tr><td rowspan="6">SELECT POPULATION II</td><td rowspan="6">Identify subpopulation: Defines infected cells within the total population</td><td>Number of Leishmania – per cell:</td><td>>0</td></tr>
<tr><td>Output Population:</td><td>Infected macrophages</td></tr>
<tr><td>Number of Leishmania – per cell:</td><td>>=2</td></tr>
<tr><td>Output Population:</td><td>Double infected macrophages</td></tr>
<tr><td>Number of Leishmania – per cell:</td><td>>0</td></tr>
<tr><td>Output Population:</td><td>Seriously infected macrophages</td></tr>
<tr><td rowspan="2">DEFINE RESULTS</td><td rowspan="2">Readout values: Calculates readout values and measures the biological effects visible in the input image</td><td>Method:
Population:
Population:
Population:
Population:</td><td>List of outputs
Macrophages - number of objects
Leishmania - number of objects
Infected macrophages - number of objects
Seriously infected macrophages - number of objects</td></tr>
<tr><td>Method:
Formula:
Population Type:
Variable A: number of objects
Variable B: number of objects
Output name:</td><td>Formula Output
1. a/b 2. to 4. a/b*100
Objects
1. Leishmania
2. Infected macrophages
3. Seriously infected macrophages
4. Double infected macrophages
Macrophages
1. Leishmania per infected macrophage
2. % infected macrophages
3. % seriously infected macrophages
4. Double infected macrophages</td></tr>
</table>

Table S2: Structures of *Eh*PIb compounds and IC_{50} determination.

Compound	*Leishmania ssp.*	IC50 [95% CI]	r²
**Eh-1** 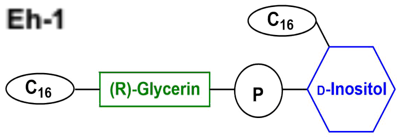	*L. major*<br>*L. braziliensis*<br>*L. donovani*<br>*L. infantum*	NA<br>NA<br>NA<br>NA	< 0.5<br>< 0.5<br>< 0.5<br>< 0.5
**Eh-2** 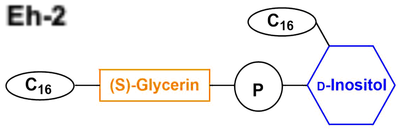	*L. major*<br>*L. braziliensis*<br>*L. donovani*<br>*L. infantum*	NA<br>NA<br>NA<br>NA	< 0.5<br>< 0.5<br>< 0.5<br>< 0.5
**Eh-3** 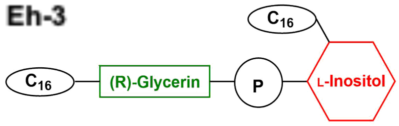	*L. major*<br>*L. braziliensis*<br>*L. donovani*<br>*L. infantum*	NA<br>NA<br>NA<br>NA	< 0.5<br>< 0.5<br>< 0.5<br>< 0.5
**Eh-4** 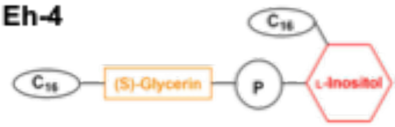	*L. major*<br>*L. braziliensis*<br>*L. donovani*<br>*L. infantum*	NA<br>NA<br>NA<br>NA	< 0.5<br>< 0.5<br>< 0.5<br>< 0.5
**Eh-5** 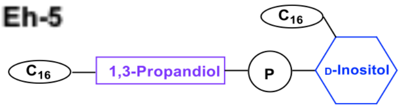	*L. major*<br>*L. braziliensis*<br>*L. donovani*<br>*L. infantum*	NA<br>NA<br>0.93 [0.48 to 1.48]<br>NA	< 0.5<br>< 0.5<br>0.9260<br>< 0.5
**Eh-6** 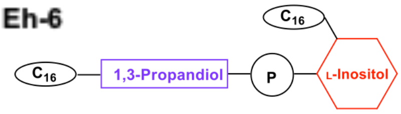	*L. major*<br>*L. braziliensis*<br>*L. donovani*<br>*L. infantum*	NA<br>4.685 [2.91 to 7.16]<br>2.294 [1.20 to 3.73]<br>NA	< 0.5<br>0.5929<br>0.7778<br>< 0.5

Abbreviations: NA = not available, CI = confidence interval

Supplementary figures

A

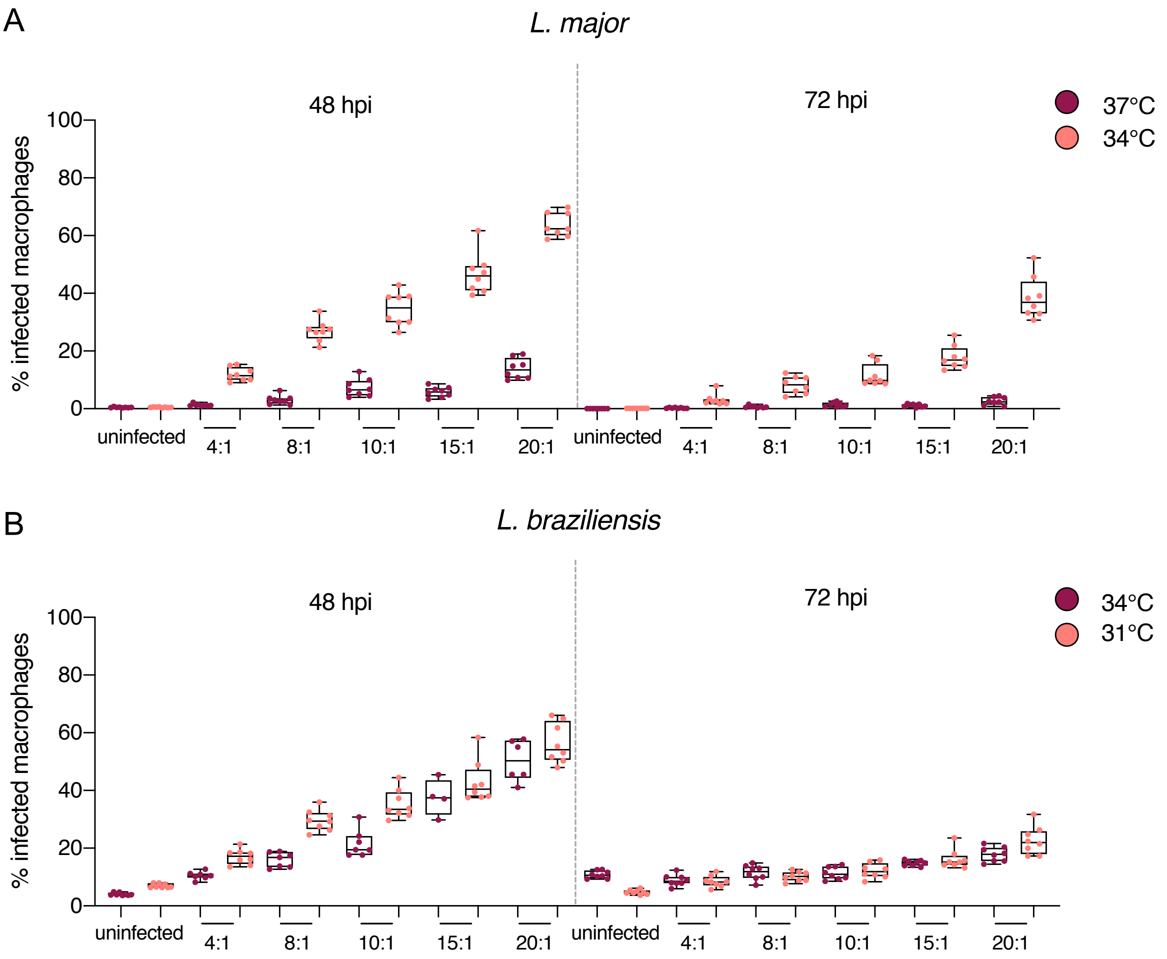

Figure S1: Influence on assay temperature for infection with cutaneous *Leishmania* species.

Percentage of infected bone-marrow derived macrophages (BMDMs) infected with (**A)** *L. major* (37°C, 34°C) or (**B)** *L. braziliensis* (34°C, 31°C) at different incubation temperatures and different multiplicities of infection (MOIs; 4:1/8:1/10:1/15:1/20:1) after 48 h and 72 h post infection. Data is expressed as boxplots with whiskers from minimum to maximum of percent of infected macrophages (n=8).
